# British and American Hospitals

**Published:** 1906-08-04

**Authors:** William Epps

**Affiliations:** Secretary, Royal Prince Alfred Hospital, Sydney.


### BRITISH AND AMERICAN HOSPITALS.

### FROM AN AUSTRALIAN STANDPOINT. By William Epps, Secretary, Royal Prince Alfred Hospital, Sydney.

(*Continued from page* 273.)

For the sake of comparison I have drawn up a table from the reports of various institutions I visited, showing the amounts derived from the contributions of patients and from donations or subscriptions, as compared with the whole incomes, as follows:—

It is, indeed, curious that in democratic America there should be these sharp distinctions, while in Great Britain, with its supposed class boundaries, all patients should be upon one dead level. Of course, it has to be remembered that in America the paying patients generally pay for all they get, and a

| Hospital. | Occupied beds. | Revenue. | | | Total expenditure. |
|---|---|---|---|---|---|
| | | From patients. | From donations. | Total. | |
| U.S.A. | | Dollars. | Dollars. | Dollars. | Dollars. |
| Presbyterian, Chicago ... ... ... ... ... ... | 181 | 87,644 | 16,872 | 184,971 | 197,182 |
| St. Luke's, Chicago ... ... ... ... ... ... | 144 | 58,116 | 6,867 | 93,347 | 90,553 |
| North-Western, Minneapolis ... ... ... ... | 50 | 22,826 | 341 | 28,327 | 27,362 |
| Massachusetts Homœopathic, Boston ... ... ... ... | 190 | 73,264 | — | 132,314 | 145,620 |
| Grace, Detroit ... ... ... ... ... ... ... | 74 | 31,827 | — | 60,553 | 61,202 |
| Harper, Detroit ... ... ... ... ... ... ... | 119 | 60,974 | 756 | 83,404 | 80,928 |
| General, Buffalo ... ... ... ... ... ... ... | 151 | 53,577 | 186 | 78,390 | 83,697 |
| Presbyterian, New York ... ... ... ... ... ... | 196 | 52,515 | 42,059 | 167,533 | 213,356 |
| John's Hopkins, Baltimore ... ... ... ... ... | 276 | 64,455 | — | 249,771 | 200,215 |
| Roosevelt, New York ... ... ... ... ... ... | 182 | 102,572 | 10,242 | 180,629 | 182,779 |
| French Hospital, New York ... ... ... ... ... | 50 | 15,429 | 3,992 | 25,770 | 25,111 |
| St. Luke's, New York ... ... ... ... ... ... | 209 | 53,255 | 17,535 | 186,445 | 186,445 |
| Mount Sinai, New York ... ... ... ... ... ... | 220 | 58,585 | 115,147 | 227,332 | 229,646 |
| Lakeside, Cleveland ... ... ... ... ... ... | 166 | 53,332 | 29,975 | 139,267 | 139,267 |
| CANADA. | | | | | |
| Vancouver General ... ... ... ... ... ... | 55 | 8,995 | 40 | 29,588 | 31,801 |
| Winnipeg General ... ... ... ... ... ... | 206 | 30,584 | 6,009 | 86,829 | 87,231 |
| Montreal General ... ... ... ... ... ... ... | 189 | 22,335 | 34,001 | 83,589 | 95,508 |
| Royal Victoria, Montreal ... ... ... ... ... | 205 | 34,882 | 1,437 | 148,836 | 148,836 |
| Grace, Toronto ... ... ... ... ... ... ... | 72 | 15,831 | 30 | 22,364 | 23,323 |
| Jeffrey Hales, Quebec ... ... ... ... ... ... | 51 | 3,964 | 2,840 | 16,658 | 15,508 |
| Calgary General ... ... ... ... ... ... ... | 40 | 9,371 | 1,295 | 16,538 | 16,223 |

A glance at this table will show that, in the United States hospitals quoted, approximately one-half or more—often much more—of the revenue comes from patients, while but a small proportion is usually raised in the form of public subscriptions. In Canadian hospitals the proportion is not so high, but in most cases the State or municipal bodies contribute a subsidy. In the magnificent Royal Victoria Hospital at Montreal the balance of income beyond that received from the patients is understood to be contributed by those millionaire philanthropists, Lords Strathcona and Mount Stephen, or from endowments supplied by them.

In making a comparison between the conditions of the British and American hospitals, therefore, one has to bear in mind the fundamental difference in the bases of maintenance. In England, with the exceptions of Guy's and St. Thomas's in London, and the Royal Infirmary at Liverpool, which were the only British general hospitals in which I found any payments made by in-patients, all patients are free and equal. There is thus but one class of menu, one class of accommodation, and one broad line of treatment throughout.

bit over, and are not dependent upon charity in any form, except in so far as they have the benefit of the endowments, established over many years. As a matter of fact, what they pay goes to help to keep the hospitals going, and in many cases the hospitals could not be maintained otherwise, as, probably, if all patients were admitted free, the sums now raised from paying patients could not be obtained from public subscriptions. In effect, therefore, it may be said that the wealthier classes, while paying for what they receive, at the same time assist to provide hospital accommodation for their poorer brethren. I do not propose here to consider the merits or demerits of this system as compared with the all-free British plan. It is a large subject, the discussion of which I have not yet seen in these pages, and one which might be threshed out with advantage. I merely state the facts as I find them.

Perhaps it may be interesting, while on this subject of revenue, to place on record, by way of comparison, a brief statement of the Australian scheme. I do not include New Zealand, as there the method of revenue-raising differs again, and is in the nature of a hospital tax, all the revenue over and above that from patients, services, and small subscriptions being practically derived from local municipal taxes, supplemented by a Government subsidy. The Australian plan generally differs to the extent that, except in Melbourne, where a small voluntary grant is made by various municipalities, amounting in all to not much more than £1,000 a year, the revenue is provided jointly by the patients, the State, and the generous public, or by the patients and the State alone. In one instance the State provides the whole of the funds, and any contributions received from patients go towards the maintenance of the grounds, etc., and are not included in the income proper of the hospital. In one other instance the State provides a fixed grant, and the institution is practically a State hospital, though subscriptions and donations are received, and whatever income can be obtained from patients is procured; while in three others the latter conditions also prevail, and the State finds the balance of income required. For the information of readers of The Hospital I have compiled a table showing the chief features of the system of income-raising in the larger general hospitals of Australia:—

With reference to Queensland, the vote by Parliament has lately been greatly reduced, and the Brisbane Hospital, which is the only large one in the State, is feeling the pinch greatly, and has not since the reduction been able to make ends meet. The whole subject is now under discussion and a new basis may shortly be arrived at.

In New South Wales the Government support is given in the form of pound for pound subsidy, upon all amounts contributed by the public, up to a fixed limit, according to the size of the hospital, the maximum being £4,000 per annum in the case of the Sydney and Royal Prince Alfred Hospitals. As will be observed from the table given, the State has also a general hospital known as the "Coast," which originally came into existence owing to an outbreak of small-pox, and has since developed into a hospital with separate infectious quarters besides about 200 "general" beds. The latter are filled with cases of persons who can afford, as a general rule, to contribute nothing to the cost of their maintenance, and they constitute the less acute medical and less important surgical cases amongst the destitute sick.

| Hospital. | Average occupied beds. | Revenue. | | | | Expenditure. |
|---|---|---|---|---|---|---|
| | | State. | Patients. | Subs. and donations. | Total. | |
| | | £ | £ | £ | £ | £ |
| Brisbane, Queensland ... ... ... ... ... | 242 | 7,583 | 1,261 | 4,201 | 16,441 | 15,677 |
| Sydney, New South Wales ... ... ... ... ... | 301 | 18,702 | 2,079 | 3,349 | 29,792 | 28,010 |
| Royal Prince Alfred, Sydney, New South Wales ... | *230 | 13,749 | 2,432 | 3,382 | 21,119 | 21,092 |
| Coast Hospital, Sydney, New South Wales ... ... | †305 | 20,997 | — | — | 20,997 | 20,997 |
| Newcastle, New South Wales ... ... ... ... ... | 60 | 1,019 | 1,068 | 1,412 | 4,520 | 4,405 |
| Melbourne, Victoria ... ... ... ... ... ... | 296 | 10,000 | 2,854 | ‡6,827 | 23,200 | 25,027 |
| Alfred, Melbourne, Victoria ... ... ... ... ... | 163 | 4,000 | 2,138 | ‡4,134 | 11,952 | 10,846 |
| Bendigo, Victoria ... ... ... ... ... ... ... | 109 | 2,600 | 526 | ‡2,190 | 6,424 | 6,713 |
| Ballarat, Victoria ... ... ... ... ... ... ... | 105 | 2,400 | 293 | ‡1,587 | 4,488 | 5,143 |
| Geelong, Victoria... ... ... ... ... ... ... | 156 | 2,400 | 610 | ‡1,599 | 6,523 | 5,767 |
| Adelaide, South Australia ... ... ... ... ... | §214 | 13,510 | 799 | 915 | 15,491 | 15,491 |
| Perth, West Australia ... ... ... ... ... ... | §130 | 10,000 | 1,150 | 1,084 | 12,411 | 12,271 |
| Hobart, Tasmania... ... ... ... ... ... ... | §89 | 5,682 | — | 1,288 | 7,648 | 7,648 |
| Launceston, Tasmania ... ... ... ... ... ... | §94 | 6,066 | Miscellaneous | 1,110 | 7,176 | 7,176 |
| Totals ... ... ... ... ... ... ... | 2,494 | 118,708 | 15,210 | 31,668 | 188,182 | 186,263 |

* Available beds now 480. † State hospital purely. ‡ Including municipal grants. § Practically a State hospital, and under State and official control.

These figures show, what I have previously indicated, namely, that the State plays a very prominent part in the finances of all the large general hospitals. (By this I mean the local State Governments and not the Commonwealth Government.) Practically the State provides two-thirds of the income. The conditions in the various States differ somewhat, but the general result is the same in Queensland, N.S.W., and Victoria. In each of these States a definite sum is received from the State, and the hospital managers have to find the rest, while nominally the same applies in West Australia, though in the latter case the amount received from the State is apparently ample to carry on the hospital together with what usually comes from other sources. In South Australia and Tasmania the State is responsible for all the necessary revenue over and above what happens to come from patients and the public. In the absence of a poor-law in Australia, the State has thus to provide for those indigent persons who would, in Great Britain, go to a poor-house infirmary, and in Sydney the demands of this class far exceed the accommodation at the Coast Hospital. As a means of avoiding the erection of a new State hospital, the State Government had for many years an arrangement with the two large general hospitals in Sydney—the Sydney and Royal Prince Alfred Hospitals—by which all the overplus general cases from the Coast Hospital, generally the most important medically and surgically, have been sent to those hospitals on a basis of payment at per diem, the amount latterly being 3s. 6d. Last year, however, this arrangement was abrogated, and the State Government has decided to give the two hospitals a special bed subsidy of £35 per bed on all occupied beds on condition that all cases sent by the Government Medical Department are to be admitted. In this way the revenue from the State has reached the proportions indicated in the table, and it will be seen that practically all the income of the hospitals from the State over and above £4,000 per annum is really in the nature of payment for the treatment of what are practically pauper patients. At the same time, the hospitals have the right to derive what income they can from the patients themselves. In Victoria the endowment is a fixed one from the State, based practically on the size of the hospital, and as there is no State general hospital, the other general hospitals have to provide accommodation for all general patients. As I have already indicated, the State is practically responsible for the maintenance of the larger hospitals in South Australia, West Australia, and Tasmania.

(*To be continued.*)

